# The influencing mechanism of scenic spot online attention and tourists’ purchase behavior: an AISAS model based investigation

**DOI:** 10.3389/fpsyg.2024.1386350

**Published:** 2024-05-23

**Authors:** Shuhong Zhao, Yingying Kong, Yueqin Yang, Jiayi Li

**Affiliations:** School of Business Administration and Tourism Management, Yunnan University, Kunming, Yunnan, China

**Keywords:** online attention, high-quality scenic spots, purchase behavior, AISAS model, Yunnan Province

## Abstract

**Introduction:**

In the era of the Internet, online digital traces have become a new way to study the online attention of scenic spots and tourists’ purchase behavior. The public’s information search on major search platforms is a series of manifestations of potential tourists’ attention and interest in scenic spots, but there are few studies on how attention, interest and information search affect potential tourists to generate real purchase behavior.

**Method:**

This paper selects four dimensions of short video platform, travel website, search engine and social media to comprehensively measure the online attention of high-quality scenic spots in Yunnan Province, and then establishes a gray association analytic hierarchy process based on the relevant variables of the AISAS model to empirically analyze the primary and secondary factors affecting tourists’ purchase behavior.

**Results:**

(1) From the perspective of the online attention of scenic spots on different platforms, the intensity of the public’s scenic spots online attention on the four types of media platforms is in the order of travel websites, search engines, short videos and social media (2) From the perspective of spatial distribution characteristics, the online attention of high-quality scenic spots in Yunnan Province is unevenly distributed, that is, there is a big difference between the attention of higher star scenic spots and their star rating and popularity, while the attention of low-star scenic spots is not much different from their star rating and popularity (3) From the perspective of spatial agglomeration characteristics, the comprehensive online attention of high-quality scenic spots in Yunnan Province presents the spatial agglomeration characteristics of “the multi-core linkage of high-density in the east and north, and sub-high-density in the south” (4) The factors influencing the purchase behavior of potential tourists are sharing experience, attracting attention, generating interest and searching information.

**Discussion:**

By exploring the formation mechanism of high-quality scenic spots online attention in Yunnan Province and the mechanism of its spatial differentiation, this study not only enriches the logical chain of “tourism information source → potential tourists → demand driven → tourism information search → travel preference → destination selection → purchase decision → travel experience → real tourists → feelings after traveling → focus on feedback → tourism information source,” but also broadens the application scenarios and application boundaries of travel preference theory and AISAS behavior model to a certain extent.

## 1 Introduction

The advancement of information and communication technologies (ICTs), particularly the Internet, has significantly impacted on the tourism sector (Amaro and Duarte, 2013). According to the China Internet Network Information Center (CNNIC) as of June 2023, the number of Internet users in China have reached 1.079 billion, and the Internet penetration rate reached 76.4%. Among them, the number of online travel bookings has reached 454 million, accounting for 42.1% of all Internet users.^1^ These increases reflect the accelerated development of the information age, which can provide new impetus for the transformation and upgrading of the tourism industry and tourism consumption.

With the most concentrated of tourism resources (Li et al., 2022), scenic spots play a crucial role in promoting the development of tourism (Chen and Wu, 2021). Gunn, a pioneer in tourist attraction research, categorizes it as (Gunn, 1993): tourist attraction, tourism support service area, and commercial service area. MacCannell (2013) argues that a tourist attraction consists of tourists, attractions, and signs. Some scholars also discuss it from the perspective of tourism supply and demand, think that scenic spots can serve as a symbol to inspire tourists to visit, and fulfill tourists’ tourism needs through their unique characteristics (Weidenfeld et al., 2010). This paper defines scenic spot as a spatial complex with a distinct geographical scope that attracts tourists to participate in tourism through exceptional scenery or human resources. Scenic spots serve as the carriers of tourists’ activities, while network media act as the “reproduction” of the actual spaces of these attractions and the “re-dissemination” of relevant information about them.

With the penetration and integration of the Internet into the tourism industry, travel websites, search engines, social media and short video platforms have gradually become the main channels for people to publish, transmit and obtain information (Vuylsteke et al., 2010; Önder et al., 2020) Potential tourists use the Internet to search for ticket prices, weather, accommodation, travel tips, and after traveling, they leave digital traces by posting travel notes and comments on travel websites, social media, short video platforms, and other platforms that is, tourism online attention (TOA). The online attention of scenic spots is mainly based on online platforms, and through specific digital traces such as views, likes, and posts, it reflects the public’s attention to scenic spots and the status of tourism demand (Pan and Fesenmaier, 2006; Bi et al., 2020), which is an important source of information for analyzing tourists’ purchase behavior (Srikanth et al., 2004). According to a study conducted by Podium, nearly 60% of consumers browse online product reviews at least weekly, and 93% think online reviews influence their shopping choices.^2^ People’s attention is limited, particularly in the information age where Internet attention is scarce. Studies have shown a link between online attention and stock buying (Barber and Odean, 2008). Moreover, the public’s focus on various online platforms can influence their purchase behavior (Dasgupta and Mondria, 2018). Yet, there has been limited focus on how the online attention of scenic spots affects the purchasing behavior of tourists under different online platforms.

Given this, this paper selects four dimensions: short video platform, travel website, search engine and social media to comprehensively measure the online attention of high-quality scenic spots in Yunnan Province, and establishes a gray association analytic hierarchy process model based on the relevant variables of the AISAS model to empirically analyze the primary and secondary factors affecting tourists’ purchase behavior. The purpose of this paper is to study the following questions: (1) the formation mechanism of online attention of scenic spots (2) the spatial distribution pattern of online attention of high-quality scenic spots in Yunnan Province, and (3) the factors influencing tourists’ purchase behavior.

Overall, this paper makes the following contributions: First, it introduces the theory of travel preference and tourism information search behavior theory to explore the formation logic behind the online attention of scenic spots, enriching the “tourism information source → demand driven → purchase decision → travel experience → focus on feedback” process. The second contribution is the establishment of a quantitative model for measuring Attention-Interest-Search-Action-Share based on the AISAS behavior model. This model investigates the primary and secondary factors of the online attention of high-quality scenic spots in Yunnan Province affecting tourists’ purchase behavior by the four types of platforms, and further explores the scope of application and economic significance of the model. This is of great significance for promoting the transformation, upgrading, and high-quality development of tourism in Yunnan Province in the digital era.

### 1.1 Online attention and tourism online attention

Attention refers to the degree of social groups’ attention to an event or people; it can reflect the internal relationship between an event and social group behavior (Girvan and Newman, 2002). Whether capturing information through search engines (Zhao et al., 2020), websites (Yang et al., 2014), social media (Park et al., 2016), or short video platforms (Du et al., 2022), users will leave corresponding digital traces (Wu et al., 2024). These digital traces can reflect behavioral preferences (Cerina and Duch, 2020) and public concerns (Cho and Hong, 2009). Since then, scholars in various fields have taken the large amount of intersected Internet search data as the data source for the research of online attention by virtue of the open, shared and interactive characteristics of the Internet (Lazer et al., 2009). Based on the stronger timeliness of network data, some scholars were the first to apply network search data to epidemiological surveillance to predict regional epidemic outbreak rates (Ginsberg et al., 2009); Subsequently, some scholars have used Internet search data to predict or analyze unemployment rates, room demand (Carneiro and Mylonakis, 2009), stock investment (Kristoufek, 2013), suicide rate (Solano et al., 2016), and commodity attributes (Yakubu and Kwong, 2021).

The online attention can reflect the popularity in the process of information dissemination in time manner, while the tourism online attention is an indirect manifestation of the travel preferences of potential tourists and the actual tourist flow of scenic spots (Guanghai and Hongying, 2022). In recent years, some scholars have used search engine data as the data source to study tourism online attention (Bangwayo-Skeete and Skeete, 2015), which verified a positive correlation between tourism online attention and actual tourist flow (Huang et al., 2017); Scenic spot managers can enhance their value-added services by utilizing the public’s access data on travel websites, thus encouraging tourists to make repeat purchases (Lexhagen, 2004). Similarly, a good online travel experience can directly or indirectly change potential tourists’ attitudes, behaviors and actual intentions toward a destination (Skadberg et al., 2005). Some scholars have also found that more and more post-travel tourists like to share their travel experiences and feelings on different online platforms during or after their trips, so as to provide reference for others’ travel decisions (Pop et al., 2022). Potential tourists, on the other hand, are more inclined to obtain relevant travel information released by post-trip tourists through online channels such as search engines, social media, official websites, and short video platforms before traveling, so as to make corresponding travel plans (Pan and Fesenmaier, 2006). However, in the field of tourism, studying the online attention of scenic spots is limited to integrate various of network platforms’ data. This paper introduces the travel preference theory (TP) and tourism information search behavior (TIS) model to analyze the distribution characteristics and differences of online attention of scenic spots from a network perspective, aiming to understand tourists’ attention preferences promptly.

### 1.2 Travel preference and tourism information search

Preference first belongs to the category of economics, and it essence is the expression of attitudes or tendencies expressed by the public (Cacioppo et al., 1994). In tourism psychology, attitude is fundamental for generating preferences. Tourists develop attitudes through the “cognition-evaluation” process of specific objects, leading to the formation of travel preferences (TP). Travel preference refers to a psychological tendency presented by potential or actual tourists with regard to a certain tourism destination (Ankomah et al., 1996). Some scholars have pointed out that the first condition for tourists to choose a destination is to generate demand motivation (Um and Crompton, 1991), and tourists’ preferences play a decisive role in the choice of destinations to a greater extent (Goodall, 1991), which is an important factor influencing the travel behavior of potential tourists (Clawson and Knetsch, 2013). As a powerful platform for the sale of tourism products and services (Abou-Shouk et al., 2013), tourists can meet their travel needs by searching for relevant tourism information on the Internet (Buhalis, 1998). Information search (IS) is defined as the decision-making process in which consumers actively obtain and integrate information from various channels before making buying decisions (Schmidt and Spreng, 1996), while tourism information search (TIS) is the process of collecting and collating information about tourist destinations on major platforms before potential tourists make travel decisions (Chiang et al., 2005). Potential tourists can not only reduce travel uncertainty (Gursoy and McCleary, 2004) but also use it as a reference for destination selection and trip planning (Jenkins, 1978), thereby enhancing the travel quality. Although online platforms aggregating the information of travel-related products and services positively have a positive impact on tourists’ purchasing behavior by influencing their travel preferences, the huge amount of travel information makes it difficult for potential tourists to accurately search for the information they need (Buhalis and Law, 2008). This study analyzes the public’s search behavior on four online platforms: short video platforms, travel websites, search engines, and social media, and explores the variations in attention to scenic spots across different platforms. Furthermore, the paper introduces the AISAS behavior model to examine the impact of attention, interest, information search, and sharing experiences share on tourists’ purchasing behavior.

### 1.3 AISAS behavioral model

The predecessor of the AISAS model is the AIDMA model. The AIDMA model was proposed by the famous American advertising scientist E.S. Lewis in 1898 and systematically organized by Hall in the 1920s (Hall, 1926). The AIDMA model focuses on consumer purchasing behavior under the traditional marketing model, while the AISAS model focuses on describing consumer purchasing behavior in the Internet era. In the era of information explosion, consumers can not only search for product-related information before purchasing but also share information after using, influencing the purchasing behavior of potential consumers (Xue et al., 2021). AISAS comprises five phases: Attention, Interest, Search, Action, and Sharing. The AISAS behavior model emphasizes the significance of search and sharing processes, as they can boost purchase frequency. With the increasing integration of the Internet and the tourism sector, travelers now rely on blogs, social media, travel websites, and other platforms to seek information and buy tourism products and services. Some scholars have highlighted the importance of information search prior to travel purchases by studying online shopping behavior (Lee et al., 2007), and some have noted positive correlations between attention and interest, interest and search, and search and behavior (Hendriyani et al., 2013). Despite the gradual application of the AISAS behavior model in tourism and hotel studies (Xue et al., 2021), there remains a gap in research on the online attention of scenic spots and tourists’ purchase behavior. Therefore, this study introduced relevant variables of the AISAS model and utilized the gray correlation analytic hierarchy process to investigate the impact of differences in online attention across the four platforms on tourists’ purchasing behavior from the perspective of online attention. The findings of this research offer valuable insights for both theoretical and practical purposes. Practically, the results pinpoint primary and secondary factors that capture attention, spark interest, facilitate information search, and encourage experiences share, influencing tourists’ purchasing behavior. This enables scenic spot managers to tailor marketing strategies for each platform, thereby enhancing exposure and attracting potential tourists.

Next, section 2 presents the research design, while section 3 discusses the empirical results. Section 4 concludes, noting the study’s limitations and the directions for future research.

## 2 Materials and methods

### 2.1 Construction of evaluation indicators

#### 2.1.1 Online attention index construction

Referring to the literature on online attention (Yang et al., 2015; Park et al., 2016; Chang et al., 2020), this study divides its evaluation model into three index layers from top to bottom as follows: (1) Layer A is the comprehensive evaluation system (2) Online attention to A-level scenic spots in Yunnan Province is divided into four target layers, B_1_–B_4_ (3) Based on the criterion layer, the four indicators are further subdivided into three-level index layer, C_1_–C_13_ (Table 1).

**Table 1:** Evaluation index system of online attention.

System of index	Objects	Index	Units	Attribute	Weights
Online attention (A)	Short video platform play volume (B_1_)	TikTok (C_1_)	Times	+	0.0666
		Kuaishou (C_2_)	Times	+	0.0577
	Travel website reviews (B_2_)	Ctrip (C_3_)	Times	+	0.0673
		Dianping (C_4_)	Times	+	0.0605
		Qunar (C_5_)	Times	+	0.0719
		Fliggy (C_6_)	Times	+	0.0755
		Mafengwo (C_7_)	Times	+	0.1577
	Search engine search volume (B_3_)	Baidu (C_8_)	Times	+	0.0508
		Bing (C_9_)	Times	+	0.0079
		360 (C_10_)	Times	+	0.1669
		Sogou (C_11_)	Times	+	0.0710
	Social media reading (B_4_)	WeChat (C_12_)	Times	+	0.0210
		Weibo (C_13_)	Times	+	0.1250

#### 2.1.2 Selection of impact indicators

Scenic spots play a crucial role in the development of tourism (Pigram, 1983), with those rated 3A and above level serving as the representatives of the attractions’ brand image (Huang et al., 2010). This study adopts China’s “Delineation and Evaluation of Quality Grades of scenic spots” to define high-quality scenic spots as those 3A and above level. Through a review of the literature, it is evident that the number of likes on social media posts reflects people’s conscious attention (Oliveira et al., 2022), the average likes on short videos indicate the popularity of the content (Zhang and Fan, 2023), search engine queries represent users’ information-seeking behavior to fulfill specific needs (Wilson, 2000), and online reviews can influence consumer purchasing decisions (Wei and Lu, 2013). As a result, this study focuses on 471 high-quality scenic spots in Yunnan Province, analyzing the online search data from major platforms between January 1, 2017, and December 31, 2022. Using the AISAS behavior model, the study sets purchase behavior data as the reference sequence, while attention, interest, information search, and sharing experience data are set as the comparison sequence to explore their impact on the reference sequence. Specifically, purchase behavior is characterized by tourist numbers, attention by social media reading, interest generation by TikTok likes, information search by search engine search volume, and sharing experience by travel website reviews (Table 2).

**Table 2:** Construction of impact indicators of tourists’ purchase behavior under AISAS model.

Data series	Target layer	System layer	Indicator layer
Reference sequence	Action	The scale of the tour	Number of domestic tourists
Compare sequences	Attention	Social media reading	The number of WeChat article reads The number of Weibo topic reads
	Interest	Short video platform play volume	TikTok likes
	Search	Search engine search volume	Baidu search volume Bing search volume 360 search volume Sogou search volume
	Share	Travel website reviews	Ctrip of reviews Dianping of reviews Qunar of reviews Fliggy of reviews Mafengwo of reviews

### 2.2 Data collection

#### 2.2.1 Scenic spots directory data

A list of A-level scenic spots in Yunnan Province, released on February 15th, 2023, was downloaded from the website of the Department of Culture and Tourism of Yunnan Province. The statistics for high-quality scenic spots in 16 regions in the province were obtained. Then, the coordinate picking tool in Baidu Maps^3^ was used to obtain the latitude and longitude coordinates of each scenic spot. Finally, ArcGIS was used to display the location of each scenic spot in space.

#### 2.2.2 Online attention data

Online attention data is mainly collected from four types of platforms: short video platforms, travel websites, search engines and social media, and the specific collection steps are as follows:

1. Search engine search volume. Step 1: Enter the names of scenic spots on Baidu, Bing, 360, and Sogou. Step 2: Set the time range, and set the retrieval date to January 1st, 2017, to December 31th, 2022. Step 3: Record the retrieval quantity for each scenic spot from the four search engines
2. Travel website reviews. Step 1: Enter the names of the 471 scenic spots into five travel websites with high domestic influence: Ctrip, Dianping, Fliggy, Qunar, and Mafengwo. Step 2: Retrieve the number of comments from January 1st, 2017, to March 31th, 2023. Step 3: Use python to crawl the total number of comments of each scenic spot. Step 4: Manually eliminate duplicate reviews or invalid reviews. Step 5: Record comments on these scenic spots on the five travel websites
3. Social media reading. Regarding WeChat articles, the first step is to search the official WeChat account for each scenic spot. The next step involves retrieving tweets with the WeChat tweet time set between January 1st, 2017, and March 31th, 2023. Step 3: Given the high volume of tweets per year, this study chooses the top three tweets from the yearly promotional posts, calculates the total number of tweets read each year, and documents them. Regarding the on-site Weibo volume of the tourist attraction, the first step is to input the name of the tourist spot in the Weibo search box; The subsequent step is to select the search category as “Location”; The final step is to visit the homepage of the searched tourist spot to observe the on-site Weibo posts dated between January 1st, 2017, and March 31th, 2023, and make a record
4. Short video platform play volume. Step 1: Enter the names of the scenic spots in the search boxes of TikTok and Kuaishou Apps. Step 2: Sequence the number of video plays. Step 3: Specify the time frame from January 1st, 2017, to March 31th, 2023, and utilize web crawling technology to gather data. Step 4: Manually screen out the top five short videos whose video content is most suitable for the scenic spot and record the corresponding data.

#### 2.2.3 Impact indicator data

As shown earlier, the number of tourists in this research is utilized to depict the tourism purchasing behavior of the reference sequence. Due to the unavailability of tourist data in high-quality scenic spots in Yunnan Province and the difficulty in obtaining it, the number of tourists in 16 regions in Yunnan Province was used for this study. The data on the purchasing behavior of the reference series is sourced from the “Yunnan Provincial Statistical Yearbook” spanning from 2017 to 2022 and the statistical bulletin of the national economic and social development of each region.

It is important to highlight that to ensure the reliability and accuracy of the research findings and maintain data consistency between the reference and comparison sequence, the study involved counting the number of high-quality scenic spots in the 16 regions of Yunnan Province. Subsequently, the comparative data on high-quality scenic spots in each prefecture and city from January 1st, 2017, to December 31th, 2022, were calculated, followed by the computation of the comparative data for all 16 regions in Yunnan Province.

### 2.3 Data processing

Since the selected network attention data comes from multiple platforms, there will be dimensional differences between the data, so the article needs to standardize the original data first. The processing steps are as follows:

First, to eliminate the influence of different index dimensions, it is necessary to use the range method (Zhang et al., 2011) to standardize the original data; namely,


(1) $$X_{ij}^{'}=\left(x_{ij}-x_{\min}\right)/\left(x_{\max}-x_{\min}\right)+0.0001,$$

(2) $$X_{ij}^{'}=\left(x_{\max}-x_{ij}\right)/\left(x_{\max}-x_{\min}\right)+0.0001,$$

Where $X_{ij}^{'}$ is the original value of the *j* index of the *i* scenic spots. $x_{\max}$ and $x_{\min}$ are, respectively, the maximum and minimum values of the evaluation object under the same evaluation index. $X_{ij}^{'}$ represents the dimensionless values of different indicators; the value range is [0,1].

Second, based on the standardization of the data, the entropy method is used to determine the weight of each index according to the variation degree of each index (Wang et al., 2019). This method can avoid the influence of subjective factors on the weight of each index and can better explain the results. The steps are as follows:

Index proportion adjustment:


(3) $$P_{ij}=\frac{X_{ij}^{'}}{\overset{m}{\underset{i=1}{\sum }}X_{ij}}.$$

Index entropy value:


(4) $$E_{j}=-\frac{1}{\ln m}\overset{m}{\underset{i=1}{\sum }}P_{ij}\times \ln P_{ij}.$$

Indicator information utility value:


(5) $$D_{j}=1-E_{j}.$$

Index weight:


(6) $$W_{j}=\frac{D_{j}}{\overset{n}{\underset{j=1}{\sum }}D_{j}}.$$

Then, the comprehensive online attention index of scenic spots is calculated


(7) $$T_{\left(i\right)}=\overset{13}{\underset{j=1}{\sum }}W_{j}\times X_{ij}^{'},$$

Where $T_{\left(i\right)}$ represents the comprehensive score for online attention to i scenic spots. $W_{j}$ represents the weight value of the j index. $X_{ij}^{'}$ represents the normalized value of the i scenic spots and the j index. j indicates the number of indicators, and 1<j<13 . i indicates the number of scenic spots, and 1≤i≤471 .

### 2.4 Research method

#### 2.4.1 Nearest-neighbor index

The nearest-neighbor index can intuitively reflect the distribution types of point elements in geographical space (Mansour, 2016). The sample group comprises 471 high-quality scenic spots, and these scenic spots are distributed in a point form in geographical space. Thus, this study uses the nearest-neighbor index R to judge the spatial distribution types. If R<1 , the distribution reflects aggregation; the closer it is to 0, the higher the aggregation degree. R=1 indicates random distribution. R>1 means uniform distribution (Zhong et al., 2021):


(8) $$R=\frac{\bar{r}}{r_{E}},$$

(9) $$r_{E}=\frac{1}{2\sqrt{n/s}},$$

Where R represents the nearest-neighbor index, $r_{E}$ represents the theoretical nearest-neighbor distance, $\bar{r}$ represents the average nearest-neighbor distance, n indicates the number of scenic spots, and s is the total area of the study area.

#### 2.4.2 Kernel density estimation

Kernel density estimation judges the areal agglomeration or dispersion of point elements by counting the number of point elements in a certain neighborhood and showing the spatial distribution law of point elements in a visual form (Cong et al., 2014). This study uses the kernel density tool in ArcGIS to calculate the point vector data of online attention to scenic spots. The formula is:


(10) $$f_{h}\left(x\right)=\frac{1}{nh}\overset{n}{\underset{i=1}{\sum }}k\left(\frac{x_{i}-x}{h}\right),$$

Where $f_{h}\left(x\right)$ is the density function, k(⋅) is a kernel function, n represents the number of points in the neighborhood, h is the search radius, and $x_{i}-x$ indicates the x distance from the $x_{i}$ scenic spots.

#### 2.4.3 Gray relational analysis

Gray correlation analysis is a method to reflect the strength, size, and order of each factor by determining the size of the gray correlation value of the reference data column and several comparison data columns (Peng et al., 2023). Based on the AISAS behavior model, this paper sets the data of purchase behavior as the reference sequence $Y_{0}$ , and sets the data series of Attention $X_{1}$ , Interest $X_{2}$ , Search $X_{3}$ and Sharing $X_{4}$ experience as the comparison sequence. Therefore, the gray correlation model is used to analyze the correlation between the comparison sequence and the reference sequence, and the larger the gray correlation degree, the higher the correlation level between the comparison sequence and the reference sequence, that is, the more significant the influence of the comparison sequence on the reference sequence. The calculation steps are as follows (Zhang et al., 2023):

Determine the reference sequence $Y_{0}$ and comparison sequence $X_{i}$ for the analysis:


(11) $$\begin{array}{l} Y_{0}=\left\{Y_{0}\left(t\right),\left(t=1,2,3,\ldots 471\right)\right\},\\ X_{i}=\left\{X_{i}\left(t\right),\left(i=1,2,3,4\right)\left(t=1,2,3,\ldots 471\right)\right\}. \end{array}$$

Quantification of sequence matrices without program:


(12) $$Y_{0}^{'}=Y_{0}\left(t\right)/Y_{0}\left(1\right),X_{i}^{'}=X_{i}\left(t\right)/X_{i}\left(1\right).$$

Calculate the difference between the dimensionless reference sequence and the comparison sequence:


(13) $$\Delta_{0i}\left(t\right)=\left|Y_{0}^{'}\left(t\right)-X_{i}^{'}\left(t\right)\right|.$$

Determine the maximum and minimum values of the difference between the reference series and the comparison series:


(14) $$\Delta_{\max}=maxmax\left(\Delta_{0i}\left(t\right)\right),\Delta_{\min}=minmin\left(\Delta_{0i}\left(t\right)\right).$$

Calculate the gray correlation coefficient:


(15) $$\varepsilon_{0i}\left(t\right)=\frac{\Delta_{\min}+\rho \Delta_{\max}}{\Delta_{0i}\left(t\right)+\rho \Delta_{\max}}.$$

Where ρ is the resolution coefficient, and the value range is 0.5;

To calculate gray correlation:


(16) $$\gamma_{0i}=\frac{1}{m}\overset{m}{\underset{t=1}{\sum }}\varepsilon_{0i}\left(t\right).$$

## 3 Result analysis

### 3.1 Scenic spots online attention evaluation

The obtained data are calculated by combining the entropy method with the comprehensive evaluation method, and the online attention index of 471 high-quality scenic spots in Yunnan Province is obtained. Using the natural fracture method in ArcGIS, the comprehensive online attention index and the online attention index of four subsystems (i.e., short video platforms, travel websites, search engines, social media) are graded.

#### 3.1.1 Online attention of scenic spots in 16 regions

**Figure 1:**
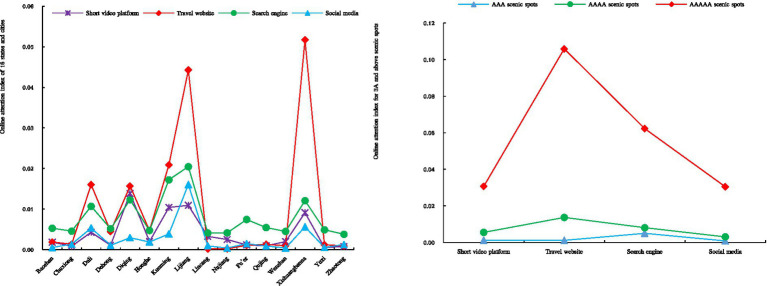
Online attention index of 16 regions and star-rated scenic spots.

It can be seen from Figure 1: Judging from the online attention of scenic spots in various regions, the public’s online attention intensity on the four types of platforms is travel websites, search engines, short videos and social media, which shows that the public is more inclined to use major travel websites to check travel destination information or share post-travel experiences, while short videos and social media play a relatively small role in attracting attention to scenic spots; despite the rapid rise of new media such as TikTok, Weibo, and Kuaishou in recent years, the vast majority of people in Yunnan Province scenic spots have not yet made full use of the powerful marketing attributes of this type of new media, and this type of new media platform has not yet truly played an intermediary role in the development of scenic spots. In addition, these four types of platforms all formed four peaks of online attention in Lijiang, Diqing, Dali, and Xishuangbanna, indicating that these regions have higher levels of attention to scenic spots. Judging from the attention of star-rated scenic spots, the public pays the highest level of attention to 5A-level scenic spots, followed by 4A and 3A-level scenic spots. This shows that the higher the star rating of a scenic spots, the higher the quality of the tourism resources in the area, and its potential for the attraction of tourists will also become stronger, which will have a positive effect on increasing the public’s online attention to high-star scenic spots.

#### 3.1.2 Online attention of each scenic spots

**Figure 2:**
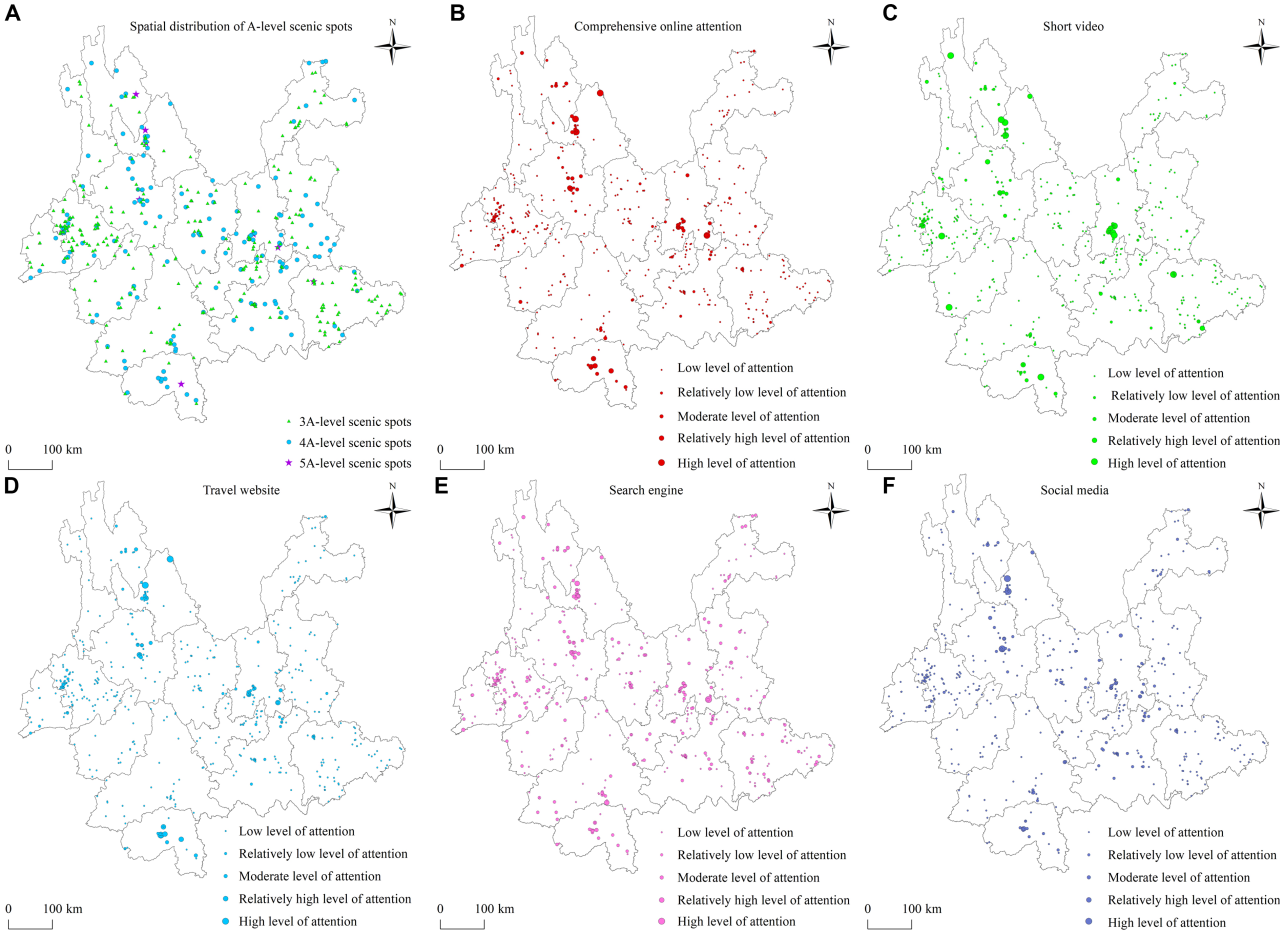
Spatial distribution map of high-quality scenic spots and their online attention in Yunnan Province (Based on the standard map no. GS (2020) 4619 of the standard map service website of the Ministry of Natural Resources; the base map boundary is not modified). **(A)** Spatial distribution of A-level scenic spots. **(B)** Comprehensive online attention. **(C)** Short video. **(D)** Travel website. **(E)** Search engine. **(F)** Social media.

Regarding the distribution of the physical and virtual spaces of scenic spots (Figure 2), there are nine 5A-level scenic spots in physical space in Yunnan Province, which are distributed in seven regions: Baoshan, Dali, Diqing, Kunming, Lijiang, Wenshan, and Xishuangbanna. In terms of virtual space, there are five 5A-level (55.56%) and six 4A-level (3.8%) scenic spots with high and relatively high attention levels. They are concentrated in Kunming, Dali, Lijiang, and Xishuangbanna. In addition, there are three 5A-level (33.33%) scenic spots, 21 4A-level (13.29%) scenic spots, and six 3A-level (1.97%) scenic spots in the study area. They have moderate attention levels and are scattered in northwest and southwest Yunnan. Regarding comprehensive online attention (Figure 2B), nine 5A-level scenic spots in Yunnan Province all have high attention to the virtual space, showing that their high level and high attention are consistent. There are 158 4A-level scenic spots in Yunnan Province, of which only 17.09% have moderate and above attention levels. More than half have low attention levels, and nearly a quarter have relatively low attention levels. This shows that the attention level of 4A-level scenic spots in Yunnan Province does not match their own influence and level. However, 98.03% of the 304 3A-level scenic spots have low and relatively low levels of attention, and only 1.97% have a moderate level of attention. This indicates that overall online attention to 3A-level scenic spots in Yunnan Province is not much different from their own levels. Regarding the four subsystems, the spatial distribution pattern of online attention of 3A level and above scenic spots in the subsystems of travel websites (Figure 2D) and search engines (Figure 2E) are similar to that of comprehensive online attention (Figure 2B). In addition, there is little difference in the number of scenic spots with low and relatively low attention levels in the four subsystems. More than 86% of the 4A-level scenic spots have low and relatively low attention levels, and less than 9% have high and relatively high attention levels.

Overall, it can be seen that the public’s attention to the network of scenic spots on the four platforms of travel websites, search engines, short videos and social media is different, and the online attention to high-quality scenic spots in Yunnan Province is unevenly distributed. That is, attention to scenic spots with higher star ratings are quite different from their star ratings and popularity, while attention to scenic spots with lower star ratings are not very different from their star ratings and popularity. We can see that the level of online attention to scenic spots in Yunnan Province is related to their star-rating levels and self-influence to certain extent.

### 3.2 Spatial characteristics of online attention to scenic spots

#### 3.2.1 Spatial distribution types

Based on the natural fracture method, online attention to each scenic spots is divided into five grades. Then, the spatial distribution types of scenic spots with different attention levels are calculated using the average nearest-neighbor tool in ArcGIS10.8 (Table 3). Regarding comprehensive online attention level, although the nearest-neighbor index R of scenic spots with relatively low attention levels is less than 1, but the p value fails to pass the test, showing that scenic spots with relatively low attention levels are randomly distributed. The nearest-neighbor index R of scenic spots with high/relatively high, moderate, and low attention levels are less than 1. The p test shows that scenic spots with high/relatively high, moderate, and low attention levels are in a state of spatial agglomeration. The spatial agglomeration level shows a trend of increasing with increased levels of attention. In summary, the number of scenic spots decreases with increased attention levels. Regarding the four subsystems, in the short video subsystem, scenic spots with high/relatively high, moderate, and relatively low attention levels are randomly distributed in space; only the scenic spots with low attention levels are clustered in space. In the travel website subsystem, the nearest-neighbor index R of scenic spots with high/relatively high, moderate, relatively low, and low attention levels is less than 1. The p test shows that the spatial structure type of scenic spots with different attention levels has always been clustered. The spatial clustering type of the search engine subsystem is the same as that of the social media subsystem. Scenic spots with high/relatively high and moderate attention levels are randomly distributed in space, and those with relatively low and low attention levels are clustered. In addition, the spatial agglomeration of scenic spots where the social media subsystem is at low and relatively low attention levels is stronger than that of the search engine subsystem

**Table 3:** Nearest-neighbor index of online attention to high-quality scenic spots in Yunnan Province.

Type	Attention level	Scenic spots number	$r_{E}\left(km\right)$	$\bar{r}\left(km\right)$	R	Z	P	Distribution type
Online attention	High/relatively high	11	94.628	36.025	0.380701	−3.929412	0.000085	Aggregated
	Moderate	30	57.300	41.417	0.722808	−2.904501	0.003678	Aggregated
	Relatively low	52	43.523	39.103	0.898447	−1.400956	0.161227	Random
	Low	378	16.143	12.977	0.803875	−7.294721	0.000000	Aggregated
Short video platform	High/relatively high	19	72.001	66.772	0.927368	−0.605673	0.544732	Random
	Moderate	14	83.879	95.645	1.140268	1.004044	0.315357	Random
	Relatively low	41	49.015	46.892	0.956688	−0.530559	0.595724	Random
	Low	397	15.752	12.119	0.769413	−8.789443	0.000000	Aggregated
Travel website	High/relatively high	10	99.247	53.559	0.539656	−2.784925	0.005354	Aggregated
	Moderate	18	73.974	33.430	0.451907	−4.448580	0.000009	Aggregated
	Relatively low	41	49.015	34.568	0.705250	−3.610583	0.000306	Aggregated
	Low	402	15.653	12.522	0.799970	−7.672538	0.000000	Aggregated
Search engine	High/relatively high	5	14.036	18.803	1.339649	1.452936	0.146241	Random
	Moderate	17	76.119	69.334	0.910864	−0.703086	0.482002	Random
	Relatively low	162	24.658	19.356	0.784975	−5.235741	0.000000	Aggregated
	Low	287	18.526	15.638	0.844113	−5.052228	0.000000	Aggregated
Social media	High/relatively high	4	15.692	16.592	1.057361	0.219472	0.826283	Random
	Moderate	13	87.045	83.866	0.963478	−0.251916	0.801106	Random
	Relatively low	71	37.247	26.909	0.722452	−4.474023	0.000008	Aggregated
	Low	383	16.037	13.073	0.815212	−6.918384	0.000000	Aggregated

In summary, because the number of star-rated scenic spots decreases with increased attention levels, the spatial density of 3A-level scenic spots is much higher than that of 4A and 5A-level scenic spots. This reflects the fact that scenic spots with low attention levels have always been clustered in space, whereas those with high/relatively high and moderate attention levels are mostly randomly distributed.

#### 3.2.2 Spatial agglomeration characteristics

**Figure 3:**
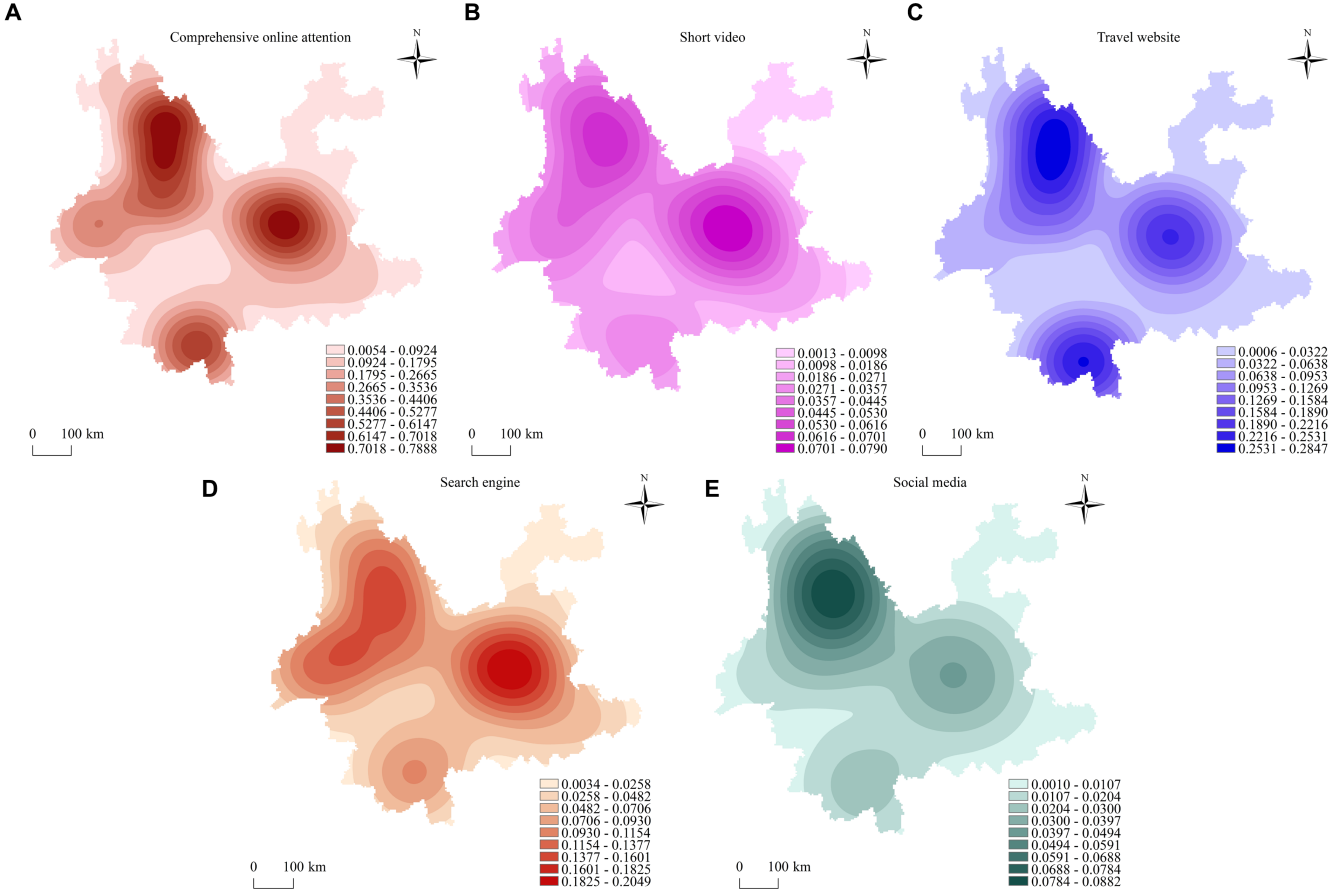
Spatial distribution map of the core density of online attention to high-quality scenic spots in Yunnan Province. **(A)** Comprehensive online attention. **(B)** Short video. **(C)** Travel website. **(D)** Search engine. **(E)** Social media.

To further explore the spatial concentration of online attention to high-quality scenic spots in Yunnan Province, this study explores the concentration of online attention to each scenic spots using kernel density analysis in ArcGIS. Regarding comprehensive online attention (Figure 3), the high-quality scenic spots in Yunnan Province have the spatial agglomeration characteristic of multicore linkage with high density in the east and north and sub-high density in the south. In the east, Kunming is the high-density core area, spreading to Chuxiong, Qujing, Yuxi, and other neighboring regions. Among them, only Kunming has two high-level scenic spots—namely, Kunming World Expo Park and Kunming Shilin. Kunming’s popularity, transportation advantages, and location advantages help achieve linkages with the surrounding scenic spots, which is also the main reason why Kunming is a high-density core area. The online attention level in the north is characterized by spatial agglomeration, with Lijiang as the high-density core area; it decreases in the direction of neighboring regions such as Dali, Diqing, and Nujiang. The high-density gathering area of this area is much larger than that of the high-density core area in Kunming, mainly because more than 50% of the high-star-rating scenic spots are gathered in this area, including Tengchong Volcanic Rehai, Diqing Shangri-La Pudacuo, Dali Chongsheng Temple Three Pagodas, Old Town of Lijiang, and Yulong Snow Mountain. It is because of these five 5A-level scenic spots that the northern region has received a lot of attention. In the southern region, the only high-level scenic spots are Xishuangbanna Tropical Botanical Garden and China Academy of Sciences. Coupled with a lack of motivation for tourism development in Pu′er, this region has become a sub-high-density area. Regarding the four subsystems, the spatial agglomerations of scenic spots are similar in the short video platform and search engine subsystems; they all show spatial agglomeration characteristics of “one high, one relatively high, and one relatively low.” However, the spatial agglomeration of each scenic spots in the travel website subsystem is similar to that of comprehensive online attention, characterized by “two high and one relatively high.” In the social media subsystem, the scenic spots show spatial agglomeration characteristics of “one high, one low, and one relatively low.”

Generally speaking, although the concentration centers of attention are different in different subsystems, whether it is a high-density core area or low-density core area, online attention to high-quality scenic spots in Yunnan Province has formed three high-value concentration centers of nuclear density in Kunming, Lijiang, Dali and Xishuangbanna, showing a spatial distribution pattern of “three multi-circle agglomeration areas and three core linkages.” Moreover, in Zhaotong, Lincang, and Wenshan, there are weak areas of nuclear density. This shows that the core density of online attention to scenic spots varies greatly in different regions.

### 3.3 Analysis of tourists’ purchase behavior on the AISAS model

From Table 4, it can be seen that the gray correlation degree of the four indicators is in the order of sharing experience (0.999), attracting attention (0.987), generating interest (0.976) and searching information (0.61), and the gray correlation degree of these four indicators are above 0.5, indicating that these four independent variables have a great impact on tourists’ purchase behavior.

**Table 4:** The correlation degree of each factor and the size of the correlation degree are sorted.

The name of the factor	Attention	Interest	Search	Share
$\gamma_{0i}$	0.987	0.976	0.61	0.999
Sort	2	3	4	1

First, share experience. In the context of the era of the Internet, more and more post-tour tourists tend to publish travel notes or reviews on major travel websites and APPs to convey their travel feelings or satisfaction. The travelogues or reviews published by real tourists are not only an important part of the tourism information source, but also greatly affect the tourists’ purchase behavior of potential tourists. For example, under positive reviews, potential tourists will yearn for the tourist destination and thus generate travel purchase motivation, while under negative reviews, potential tourists’ travel motivation will gradually weaken with the increase of negative reviews. Therefore, “sharing experience” has the greatest impact on tourists’ purchasing behavior.

Second, attract attention. In the digital economy era where traffic is crucial, numerous niche and less popular tourist destinations have effectively captured people’s interest through the Internet. Capturing attention serves as the foundation for potential tourists to develop interest, making it essential for marketing efforts at scenic spots to first capture the attention of potential tourists. Hence, in comparison to interest, attention holds a greater influence on purchasing behavior.

Third, generate interest. With the development of the economy and the upgrading of consumption, the trend of the tourism market has gradually changed to interest-oriented personalized consumption, and potential tourists are increasingly interested in scenic spots with distinctive characteristics. Scenic spots with distinctive characteristics can stimulate the interest of potential tourists and actively generate purchasing behavior.

Fourth, search information. Potential tourists actively seek detailed information about their destination of interest. However, factors such as the timeliness and relevance of online information can influence the purchasing behavior of potential tourists. Only when the management of the scenic spot effectively promotes the tourist destination to capture the attention of potential tourists and make them interested will potential tourists discover it through their search. Therefore, the influence of “search information” on purchasing behavior is not as strong as that of “interest” and “attention.”

## 4 Conclusions and discussions

### 4.1 Research findings

“Internet + tourism” is the product of the development of the tourism industry in line with the times, and the combination of the two will not only help to enhance the popularity of scenic spots, but also increase the economic benefits of scenic spots. Based on the AISAS behavior model, along with the theory of travel preference and tourism information search, this study delves into the variances in online attention toward high-quality scenic spots in Yunnan Province across four types of platforms: short video platform, travel website, search engine, and social media. It also examines the impact intensity on tourists’ purchase behavior. The research reveals that the public’s attention intensity toward high-quality scenic spots in Yunnan Province is the highest on travel websites, followed by search engines, short videos, and social media. This pattern is attributed to the limited nature of people’s attention (March, 1994), particularly in the age of mass media, where individuals must distribute their attention across various online platforms (Lu et al., 2023), leading to areas of high concentration and attention deficits. Furthermore, scholars have noted that information search significantly influences potential tourists’ purchasing decisions (Lee et al., 2007), which are influenced by factors like travel notes or reviews on prominent travel websites and apps (Wang et al., 2023), as well as the marketing strategies of tourist destinations both online and offline. Building upon the AISAS behavior model, this study further elucidates that the factors impacting potential tourists’ purchase behavior include sharing experience, attracting attention, generating interest and searching information.

### 4.2 Theoretical contributions

**Figure 4:**
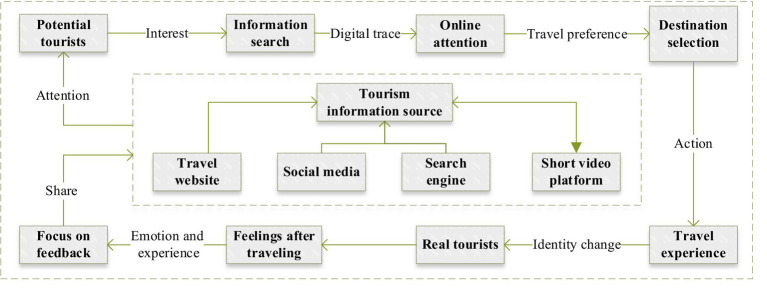
The formation mechanism of online attention under the AISAS model.

In this study, we construct a logical chain of the formation mechanism of online attention from the aspects of travel preference theory and tourism information search model. The whole process of the formation of tourism online attention is a cycle between the virtual end and the real end, that is, the potential tourists from the initial virtual online attention to the real tourism flow. First of all, in the era of big data, more and more post-tour tourists tend to publish travel notes or videos on platforms such as TikTok, Xiaohongshu, and travel websites, which are gathered by short video platforms, search engines, travel websites, social media and other platforms into a rich source of tourism information (Bangwayo-Skeete and Skeete, 2015; Park et al., 2016). Then, under a certain motivation, potential tourists generate the behavior of retrieving information about relevant scenic spots, and the digital traces left by this series of behaviors are the online attention. Then, different groups of potential tourists choose destinations according to their travel preferences (Goodall, 1991). Finally, when potential tourists have a travel experience, their identity will change from potential tourists to real tourists, and some tourists will review and share on different travel websites after the travel experience (Bilgihan et al., 2016), which can not only convey the real emotions and experiences of their trip, but also further update the tourism information source, attract the attention of potential tourists on a larger scale, and start the next round of the cycle of virtual space and real space. Therefore, this study systematically explains the formation mechanism of tourism online attention, which not only enriches the logical chain of “tourism information source → potential tourists → demand-driven → information search → travel preference → destination choice → purchase decision → travel experience → real tourists → feelings after traveling → focus on feedback → tourism information source,” but also broadens the application scenarios and application boundaries of travel preference theory and tourism information search behavior model to a certain extent (Figure 4).

### 4.3 Practical implications

The findings of this study are valuable for managers of scenic spots to utilize Internet technology in developing an effective network marketing strategy for high-quality scenic spots in Yunnan Province. The study revealed that the public pays the most attention to travel information on travel websites, indicating that the reviews on these websites have a significant impact on potential tourists’ decision to make purchases (Wang et al., 2023). For instance, managers of scenic spots can enhance the visibility and appeal of the attractions through short video platforms and social media, as well as improve the filtering and intelligent recommendation systems on travel websites to enhance the credibility of the reviews, thus encouraging potential tourists to search and ultimately make a purchase.

Furthermore, we suggest optimizing the layout of promotional videos and online reviews of scenic spots on the four platforms: short videos, travel websites, search engines, and social media, to present a wide range of hashtags to potential tourists. For example, using hashtags related to travel itineraries, transportation, hotel services, food and beverages, and cultural customs. Diverse hashtags can assist potential tourists in filtering and finding the information they need more effectively and quickly. By optimizing the layout of the platforms, tailored to the different preferences of potential tourists, we can improve their perception of the information, enhance their interest, and stimulate them to conduct more searches and improve their purchasing behavior.

### 4.4 Research limitations and suggestions for future study

This study focuses on the influencing factors of potential tourists’ purchase behavior through network data analysis, with innovative research perspectives. However, online data, as objective information, may not fully capture the impact of various platforms on potential tourists’ purchasing decisions. The study does not address whether the findings are consistent or different when considering subjective questionnaire data. Therefore, future research should incorporate questionnaire surveys to assess the influence of short video platforms, travel websites, search engines, and social media on potential tourists’ purchase behavior from both subjective and objective viewpoints. This approach will enhance the contextual relevance of the research findings and improve their accuracy. Furthermore, while data from four types of platforms were collected in large quantities, the analysis in this study only examines them statically. It fails to dynamically reflect the influence on tourists’ purchase behavior due to the complexity of their decision-making process. Hence, future research should investigate the dynamic evolution of potential tourists’ purchasing behavior by continuously tracking and supplementing data.

## Data availability statement

The raw data supporting the conclusions of this article will be made available by the authors, without undue reservation.

## Author contributions

SZ: Conceptualization, Writing – review & editing, Data curation. YK: Software, Visualization, Writing – original draft. YY: Methodology, Writing – original draft. JL: Writing – review & editing.

## Footnotes

^1^ http://finance.people.com.cn/n1/2023/0828/c1004-40065362.html

^2^ https://www.199it.com/archives/580674.html

^3^ https://api.map.baidu.com/lbsapi/getpoint/
